# New Medical School

**Published:** 1846-05-01

**Authors:** 


*New Medical School.*—Arrangements have been made for the organization of a medical school at Memphis, Tennessee. The following appointments have been made: J. Bybee, M. D., Anatomy; D. I. M. Doyle, M. D., Surgery: A. Hopton, M. D., Chemistry; G. R. Grant, M. D., Practice of Medicine. The chairs of institutes and juris-prudence,—materia-medica,—and obstetrics remain to be filled. The Trustees invite applications until July next. Communications to be addressed to R. H. Patillo, secretary of the board.

